# Mutant p53 Regulates Dicer through p63-dependent and -independent Mechanisms to Promote an Invasive Phenotype*

**DOI:** 10.1074/jbc.M113.502138

**Published:** 2013-11-12

**Authors:** Patricia A. J. Muller, Antonio G. Trinidad, Patrick T. Caswell, Jim C. Norman, Karen H. Vousden

**Affiliations:** From the ‡ Cancer Research UK Beatson Institute, Glasgow G61 1BD, Scotland, United Kingdom and; the § Wellcome Trust Centre for Cell-Matrix Research, University of Manchester, Manchester M13 9PT, United Kingdom

**Keywords:** Cell Invasion, Cell Motility, Dicer, MicroRNA, p63, Cell Scattering, Mutant p53

## Abstract

The control and processing of microRNAs (miRs) is critical in the regulation of all cellular responses. Previous studies have suggested that a reduction in the expression of certain miRs, or an overall decrease in miR processing through the partial depletion of Dicer, can promote enhanced metastatic potential. We show here that Dicer depletion can promote the invasive behavior of cells that is reflected in enhanced recycling and activation of the growth factor receptors Met and EGF receptor. These responses are also seen in response to the expression of tumor-derived mutant p53s, and we show that mutant p53 can down-regulate Dicer expression through both direct inhibition of the TAp63-mediated transcriptional activation of Dicer and a TAp63-independent control of Dicer protein expression. Our results delineate a clear relationship between mutant p53, TAp63, and Dicer that might contribute to the metastatic function of mutant p53 but, interestingly, also reveal TAp63-independent functions of mutant p53 in controlling Dicer activity.

## Teaser

**Background:** p53 mutations give rise to a mutant p53 protein that acquires novel pro-oncogenic functions.

**Results:** Mutant p53 inhibits Dicer to promote receptor recycling, which is required for invasion and scattering.

**Conclusion:** Dicer is inhibited by mutant p53 via two mechanisms: TAp63-dependent and TAp63-independent.

**Significance:** Our findings contribute to a better understanding of the proinvasive functions of mutant p53.

## Introduction

MicroRNAs (miRs)^2^ are short hairpin RNA structures that are transcribed from DNA as primary (pri)-miRs and then cleaved by Drosha to generate precursor (pre)-miRs. These pre-miRs are further processed by Dicer to generate mature miRs that can either block translation or promote the degradation of target mRNA (1). In the last decade, several studies have demonstrated an important role for miRs in various diseases, including cancer, where miRs can act as oncogenes or tumor suppressors (2). MicroRNA profiling studies demonstrated a decreased global expression of mature miRs in human and murine cancers (3), sometimes caused by decreased expression of miR biogenesis enzymes (4). In general, therefore, a failure to properly express or process miRs, for example in response to decreased Dicer expression, is associated with various aspects of malignant progression.

The p53-related proteins (comprising p53, p63, and p73) are transcription factors that regulate the expression of a large variety of miRs. p53 binding elements are present in the promoters of several microRNA processing proteins, including Dicer (5). Furthermore, murine Dicer has been shown to be a transcriptional target of TAp63 (6). p63 exists as multiple isoforms, including a full-length form with a complete transactivation (TA) domain and an N-terminally truncated form (ΔN). Studies in TA domain- or ΔN-specific knockout mice have identified distinct and separate roles for each isoform, with embryonic lethality of ΔNp63^−/−^ mice because of severe skin defects (7). TAp63^−/−^ mice are viable but display signs of obesity, aging, and a potential to form metastatic cancers (6, 8–10). Interestingly, loss of TAp63 coincided with decreased Dicer expression and a concomitant decrease in the expression of mature miRs (6). Similar results were observed in cell systems where decreased Dicer expression resulted in a down-regulation of mature miRs with a consequent promotion of extracellular matrix and invasion (11, 12).

In addition to decreased Dicer expression, many metastatic cancers acquire mutations in the tumor suppressor p53, leading to the loss of p53 or the expression of a mutant p53 protein. Mutant p53 proteins may exert a dominant negative function over wild-type p53 but also acquire novel functions to promote tumorigenesis. Although the mechanisms through which mutant p53 functions are still under investigation, some of the tumor-associated mutations promote the binding of mutant p53 to proteins such as transcription factors, transcription enhancers, or kinases. These interactions are not always seen with wild-type p53, and they allow mutant p53 to acquire an oncogenic role (13–15). Various mutant p53s can promote invasion and metastasis by interfering with TAp63 function (16, 17), although the mechanisms underlying this interference are complex. Although most p53 mutants inhibit TAp63-dependent gene expression to some extent, this does not simply correlate with the efficiency of binding of mutant p53 to TAp63 (16–22). We showed previously that the inhibition of TAp63 activity by two different p53 mutants (175H and 273H) promoted Rab coupling protein (RCP)-dependent recycling of integrins and growth factor receptors from intracellular vesicles back to the plasma membrane (16). This enhanced recycling resulted in an increase in growth factor receptor-mediated signaling and facilitated random motility, invasion, and scattering of cells (16, 23).

Various reports suggest that mutant p53 can regulate the expression of certain miRs (24–27). Notably, although some of these were regulated by mutant p53 in a p63-dependent manner (*e.g.* miR-155 and miR-205), the regulation of others (such as miR-130b) was p63-independent. Here, we explore the function of Dicer in mutant p53-driven invasion and scattering and examine the contribution of TAp63 to these activities.

## EXPERIMENTAL PROCEDURES

### 

#### 

##### Cell Culture and Constructs

H1299, HT29, MDA MB 231, and A431 cells were all purchased from the ATCC and cultured in DMEM containing 10% FBS, 1% penicillin/streptomycin, and 1% glutamine at 37 °C and 5% CO_2_. The generation of stable mutant p53 expressing H1299 cells has been described before (16). GFP and Cherry constructs were purchased from Clontech and cotransfected with an empty vector or mutant p53 following the same selection procedure. Doxycycline-inducible H1299 cells were generated as described before (28).

The generation of GFP-RCP, 273H, and 273H Δ347 has been described before (16, 29). The GNL 273H p53 construct was generated by mutagenesis using the following oligos: 5′-ACA CTG GAA GAC TCC AGT GGG AAC CTA CTG GGA CGG AAC AGC TTT-3′ (forward) and 5′-TCA AAG CTG TTC CGT CCC AGT AGG TTC CCA CTG GAG TCT TCC AGT-3′ (reverse).

##### Transfection Procedures

Knockdown in H1299 and MDA MB231 was achieved by transfection of siRNA using Hiperfect (Qiagen) according to the protocol of the manufacturer, and constructs were expressed using Genejuice (Merck-Millipore) according to the protocol of the manufacturer. Knockdown and overexpression in HT29 and A431 cells were achieved by transfection of siRNA or plasmids using the AMAXA nucleofection method, solution V, and protocols X-001 and X-005, respectively.

The following siRNA oligos were used: control siRNA 1, 5′-GCAACGGCAUUCCACCUUU(TT)-3′; control siRNA 2 control pool of four siRNAs (Dharmacon, catalog no. D-001810-10^−20^); RCP (SMARTpool of four siRNAs (Dharmacon, catalog no. L-015968-00-0005); p53, 5′-GACUCCAGUGGUAAUCUAC(TT)-3′; p63 1, 5′-UGAACAGCAUGAACAAGCU(TT)-3′; p63 2, 5′-UGACUUCAACUUUGACAUG(TT)-3′; and MET (SMARTpool of four siRNAs (Dharmacon, catalog no. L-003156-00-0005); Dicer 1, 5′-GCU CGA AAU CUU ACG CAA A (TT)-3′; and Dicer 2, 5′-CCA CAC AUC UUC AAG ACU U (TT)-3′. All siRNA oligos were used as a combination of two or four siRNAs, and each individual siRNA was tested for efficiency of knockdown and off-target effects.

For siRNA rescue experiments, MDA MB231 and HT29 cells were transfected with the AMAXA nucleofection method, solution V, and protocols X-001 and X-005 using a combination of 2 μg of plasmid and 120 pmol of p53 siRNA for 8 × 10^6^ cells.

##### Western Blot and Immunoprecipitation

For RCP immunoprecipitations, H1299 cells were transfected using AMAXA nucleofection (solution V and protocol X-001), and cells were lysed in IP lysis buffer (200 mm NaCl, 75 mm Tris-HCl (pH 7), 15 mm NaF, 1.5 mm Na_3_VO_4_, 7.5 mm EDTA, 7.5 mm EGTA, 0.15% Tween 20, 50 μg/ml leupeptin, 50 μg/ml aprotinin, and 1 mm 4-(2-aminoethyl)-benzenesulfonyl fluoride. RCP was immunoprecipitated as described previously using a GFP antibody (Roche) and magnetic protein G beads (30). Integrin immunoprecipitation was quantified from scanned films using ImageJ.

For all other Western blot procedures, cells were harvested in Nonidet P-40 lysis buffer (150 mm NaCl, 50 mm Tric-HCl (pH 8.0), and 1% Nonidet P-40) supplemented with a complete protease inhibitor tablet (Roche) and incubated for 15 min on ice. Cell debris was spun down at 4 °C for 15 min (maximum speed), and the supernatant was combined with sample buffer, boiled for 5 min at 95 °C, and run on an SDS-PAGE gel. For MET and EGFR activation assays, cells were immediately harvested in sample buffer after the indicated EGF or HGF incubation times and sheared using an insulin needle. The following primary antibodies were used: p53 DO-1 (1:5000, monoclonal (31), p53 1801 (1:5000), and pMET (Y1234/5, Cell Signaling Technology), MET (1:250, R&D Systems), pEGFR (1:1000, Sigma), EGFR (1:1000, Cell Signaling Technology), α5 integrin (1:1000, BD Biosciences), RCP (1:2000, Sigma), Rab11 (1:1000, Invitrogen), GFP (1:2000, Roche), GCN5 (1:2500, Santa Cruz Biotechnology), Dicer (1:250, Abcam), actin (1:5000, Millipore), and p63 (BC4A4, 1:500, Santa Cruz Biotechnology). Western blot analyses were quantified using Li-Cor image studio software.

##### Invasion Assays

Invasion assays were performed as described previously (16). Briefly, Matrigel (BD Biosciences) was diluted in PBS to a protein concentration of 6 mg/ml, supplemented with 25 ng/ml fibronectin (Sigma), and polymerized in transwell inserts. After polymerization, the transwells were inverted, and 2.5 × 10^4^ (H1299) or 3.5 × 10^4^ (MDA MB 231) cells were seeded on the membrane. After settling for 4–6 h, the transwells were placed in serum-free medium, and the upper chamber was filled with 100 μl of medium supplemented with 10% FBS and EGF (25 ng/ml) or HGF (10 ng/ml for H1299 and 30 ng/ml for MDA MB231). Invasion was monitored by staining the cells with 4 nm calcein and was visualized by confocal microscopy (Leica 2) in serial sections of 15 μm throughout the Matrigel plug. Invasion was quantified using ImageJ and the plugin area calculation, in which the total intensity of all slides beyond 30 μm was determined as a percentage of invasion in all slides.

##### Scattering

Scatter assays were performed as described previously (23). Cells were seeded sparsely and transfected as described elsewhere. After 6 h, H1299 cells were washed and allowed to form colonies for 48 h. HT29 cells were washed after 6 h and incubated in HGF for 72 h to induce scattering. Phase-contrast images of scattered cells were taken with an Olympus CKX41 microscope, and lysates were harvested to verify knockdown. Scattering was quantified by counting the number of “scattered” or “unscattered” cells per image in each experiment using the ImageJ plugin “cell counter,” and the proportion of scattered cells was plotted. Scattered H1299 cells were defined as cells that were growing in colonies of four cells or less. In HT29 cells, cells were defined as scattered if they were only touching one other neighboring cell.

##### Immunofluorescence

H1299 cells were grown on glass coverslips, fixed in 4% paraformaldehyde for 10 min at 4 °C, and permeabilized in 0.2% Triton X-100 in PBS for 15 min at room temperature. Cells were blocked in 1% BSA in PBS, followed by incubation in Dicer (1:50, Abcam) and ZO-1 (1:75, Cell Signaling Technology) antibodies for 1 h at room temperature. After three PBS washes, cells were incubated in secondary mouse Alexa Fluor 488 antibody for 1 h at room temperature and supplemented with DAPI (Sigma, 0.5 μg/ml). Cells were washed five times with PBS and mounted on microscope slides. Confocal images were made using a Zeiss 710 microscope.

##### Recycling Assays

Recycling assays were performed as described previously (23, 30, 32). Briefly, cells were serum-starved and biotinylated for 45 min. Biotinylated receptors were allowed to recycle back to the plasma membrane for 0, 15, or 30 min, after which the percentage of intracellular biotinylated receptors was determined using capture enzyme-linked immunosorbent assays.

##### Mature miR, Pri-miR, and mRNA Expression

RNA for detection of miR, pri-miR, or mRNA was isolated using TRIzol according to the protocol of the manufacturer. For mRNA and pri-miR detection, cDNA was generated using oligo(dT) and a first-strand synthesis kit (Invitrogen) according to the instructions of the manufacturer. For the RT-PCR reaction, 5 μl of the 40×-diluted cDNA was used in combination with 10 ml of SYBR Green Master Mix (Thermo Scientific), 1 μl of each oligo (10 μm), and 3 μl of H_2_O with the following PCR conditions: 94 °C annealing for 2 min, 40 cycles of 30 s at 94 °C, 30 s at 60 °C, and 1 min at 72 °C, followed by a 10-min 72 °C incubation. A melting curve was then generated to check for specificity of the oligos. mRNA expression of GAPDH was used as a reference. The oligos used were as follows: GAPDH, 5′-GCA GAG ATG ATG ACC CTT TTG GCT-3′ (forward) and 5′-TGA AGC TCG GAG TCA ACG GAT TTG GT-3′ (reverse); p63, 5′-TTC TTA GCG AGG TTG GGC TG-3′ (forward) and 5′-GAT CGC ATG TCG AAA TTG CTC-3′ (reverse); Dicer, 5′ AGC TGT CCT ATC AGA TCA GGG-3′ (forward) and 5′-CAT TCA AGG CGA CAT AGC AAG T-3′ (reverse); pri-miR-130b, 5′-CCT GTT GCA CTA CTA TAG GCC G-3′ (forward) and 5′-TGC CCT TTT AAC ATT GCA CTG-3′ (reverse); and pri-miR-206, 5′-ACA TGC TTC TTT ATA TCC CCA-3′ (forward) and 5′-AAA CCA CAC ACT TCC TTA CAT TC-3′ (reverse).

For the detection of mature miRs, miR-specific oligos were ordered from Applied Biosystems, and mature miR expression was determined using the 7500 fast real-time PCR machine according to the protocol of the manufacturer (Applied Biosystems).

## RESULTS

### 

#### 

##### Dicer Depletion Drives Invasion and Scattering through RCP-dependent MET and EGFR Activation

**FIGURE 1.**
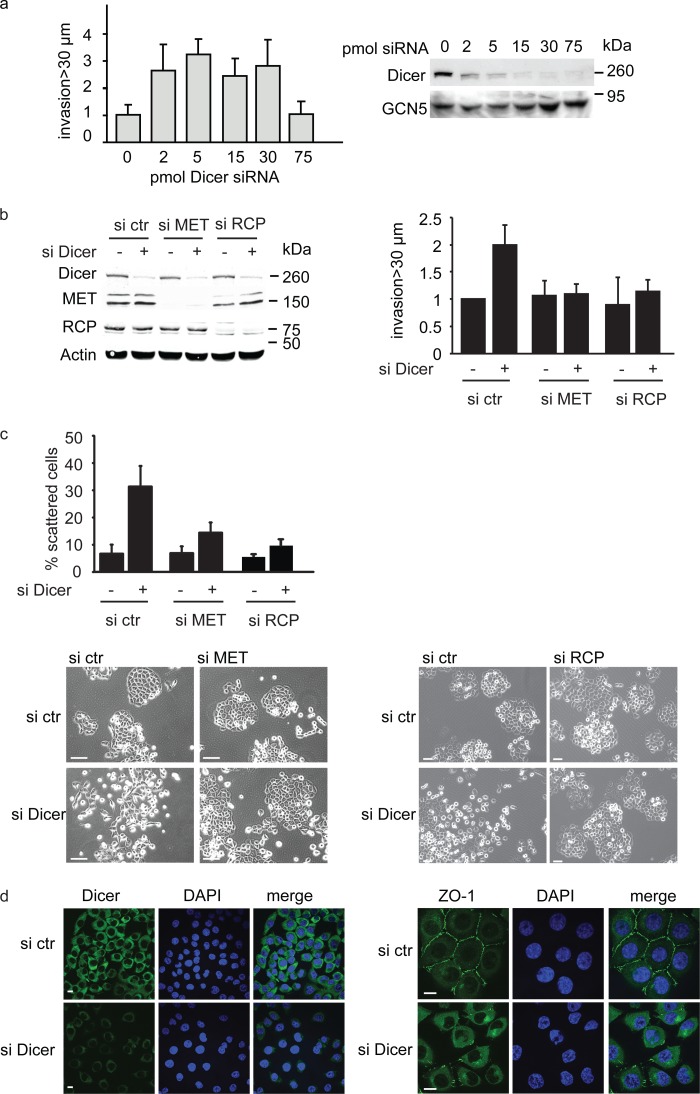
**Dicer knockdown promotes cell invasion and scattering.** *a*, H1299 cells were transfected with increasing amounts of Dicer siRNA, and invasion was measured in Matrigel invasion assays (*left panel*). Values are mean ± S.E. of five experiments. Knockdown of Dicer was verified by Western blot analysis, and a representative Western blot of one of the experiments in the graph is shown. GCN5 was used as loading control (*right panel*). See also Fig. 4*d. b* and *c*, H1299 cells were transfected with Dicer, MET, and/or RCP siRNA and monitored for invasion (*b*) or scattering (*c*). *c*, invasion is quantified in the *right panels*, and scattering is quantified in the *top panel. Error bars* indicate S.E. of three experiments. *Scale bars* = 50 μm. *ctr*, control. *d*, H1299 cells were transfected with Dicer siRNA, and Dicer expression (*left panel*, *green*) and ZO-1 (*right panel*, *green*) localization was determined by immunofluorescence. DAPI is shown in *blue. Scale bars* = 10 μm.

Several studies have demonstrated a role for Dicer in limiting tumorigenesis, with a decrease in Dicer resulting in increased invasion (11, 12, 33). We found a similar effect following incomplete siRNA-mediated depletion of Dicer in H1299 cells, which showed enhanced invasion toward HGF in response to partial Dicer depletion (Fig. 1*a*). As shown previously, this invasive capacity was lost following a more efficient knockdown of Dicer (Fig. 1*a*), probably because of sensitization of cells to apoptosis or cell death (11). To avoid artifacts caused by cell death, all experiments hereafter were performed with 15 pmol siRNA of Dicer.

**FIGURE 2.**
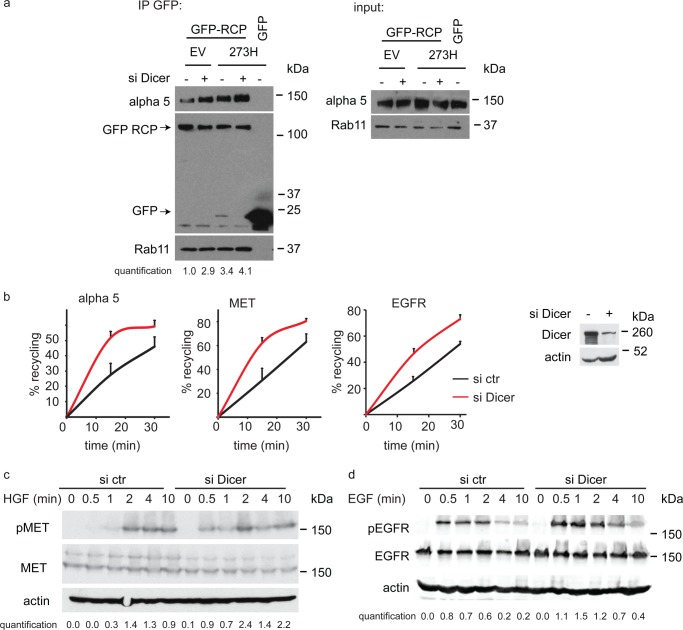
**Dicer knockdown promotes MET and EGFR recycling and signaling.** *a*, H1299 empty vector (*EV*) or mutant p53-expressing cells (*273H*) were transfected with Dicer siRNA and GFP-RCP or a GFP control. Lysates were immunoprecipitated (*IP*) with a GFP antibody, and proteins were detected by Western blot analysis using antibodies to detect α 5 integrin, GFP, or Rab11 expression. Input levels are shown in the *right panel*, and α 5 levels in the immunoprecipitates were quantified with respect to the levels of GFP-RCP present in the same samples. The quantification is shown below the blot in which α 5 levels in EV cells transfected with control siRNA were set to 1. *b*, recycling of α 5 integrin, MET, and EGFR was determined in H1299 cells transfected with Dicer siRNA. Dicer knockdown was verified by Western blot analysis using actin as loading control (*ctr*). *c* and *d*, H1299 cells were transfected with Dicer siRNA and treated with 10 ng/ml HGF (*c*) or 10 ng/ml EGF (*d*) for the indicated times. Phosphorylation was detected using phospho-specific antibodies for MET and EGFR, and total MET, EGFR, and actin antibodies were used as loading controls. pEGFR and pMET expression were quantified and corrected for EGFR or MET expression levels, as shown under each panel.

Enhanced invasion of H1299 cells toward HGF following Dicer depletion was dependent on the HGF receptor MET, as invasion was opposed by simultaneous transfection of MET siRNA with siRNAs targeting Dicer (Fig. 1*b*). MET signaling can drive cell scattering, and we found that a siRNA-mediated reduction in Dicer expression promoted scattering in a MET-dependent manner (Fig. 1*c*). Concomitant with scattering, loss of ZO-1 from the cell-cell junctions was apparent after knockdown of Dicer (Fig. 1*d*). Our previous studies showed expression of mutant p53 in H1299 cells that do not express any p53 protein endogenously, enhanced scattering and invasion by promoting MET receptor recycling that is driven by α5β1 integrin and the Rab11 effector protein RCP (23). Depletion of RCP in Dicer knockdown cells prevented invasion (Fig. 1*b*) and scattering (*c*), indicating that RCP is important in mediating invasion and scattering after reduced Dicer expression. To investigate whether Dicer can play a role in regulating RCP-driven recycling, we initially investigated the interaction between RCP and α5 integrin. Knockdown of Dicer enhanced the association between RCP and α5 integrin, whereas the known interaction between RCP and Rab11 remained unchanged (Fig. 2*a*). Mutant p53 expression also increased the RCP-α5 integrin association, consistent with our previous results (16) (Fig. 2*a*). Furthermore, we also observed enhanced recycling of α5 integrin, MET, and EGFR after knockdown of Dicer (Fig. 2*b*).

Enhanced recycling of MET results in increased activity (23), so we examined the effect of Dicer depletion on MET phosphorylation (as an indication of activation) in response to HGF stimulation (Fig. 2*c*). Although Dicer knockdown did not induce MET signaling in the absence of HGF, a reduced expression of Dicer sensitized cells so that they signaled more rapidly in response to stimulation with low levels of HGF. As seen with mutant p53-induced receptor recycling (16), the effect of Dicer depletion was not confined to MET but also resulted in enhanced recycling of the EGFR (Fig. 2*c*) and promoted a more robust EGFR activation upon ligand (EGF) stimulation (Fig. 2*d*). Together, these data demonstrate a role for Dicer in limiting receptor recycling to restrain downstream signaling, cell invasion, and scattering.

##### Mutant p53 Decreases Dicer Expression through TAp63 Inhibition

**FIGURE 3.**
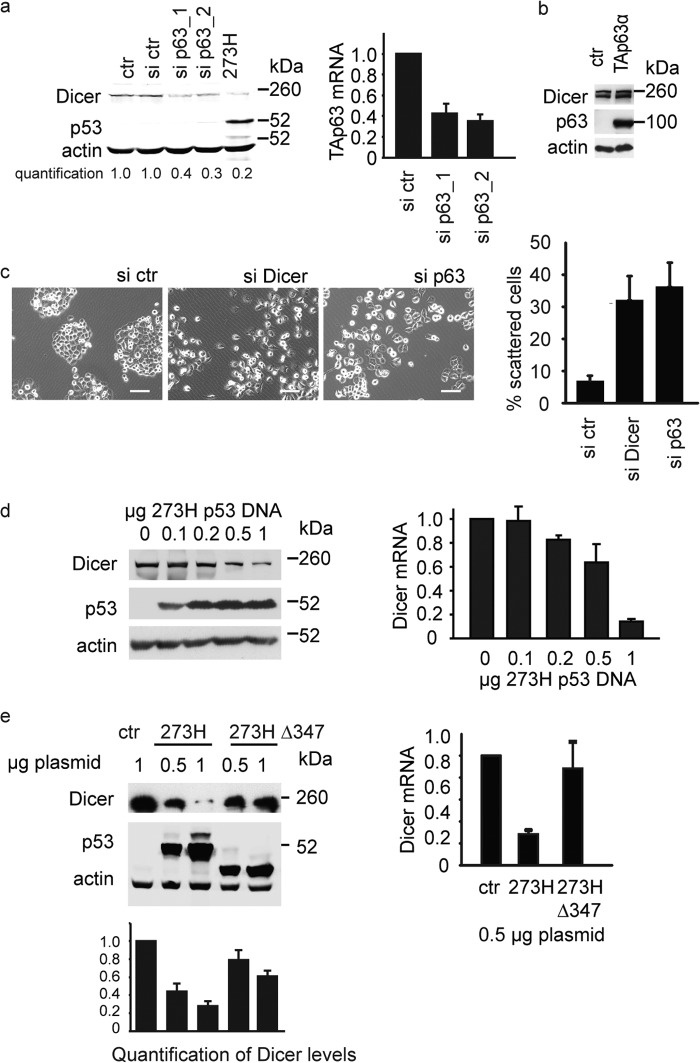
**Mutant p53 decreases Dicer expression.** *a*, H1299 cells were transfected with p63 siRNA, control siRNA (*si ctr*), an empty vector (*ctr*), or mutant p53 273H and monitored for Dicer expression using Western blot analysis. Actin was used as a loading control. Dicer expression levels were quantified and corrected for actin expression. Quantification is shown under the blot, and expression of Dicer in empty vector-transfected cells was set to 1. p63 knockdown was confirmed using qRT-PCR (*right panel*). Values are mean ± S.D. of the p63 levels corresponding to the experiment shown in the Western blot. *b*, H1299 cells were transfected with TAp63α, and Dicer and p63 expression was determined by Western blot analysis. Actin was used as a loading control. *c*, H1299 cells were transfected with Dicer or p63 siRNA and monitored for scattering (*left panel*). Scattering was quantified, and values are mean ± S.E. of three experiments (*right panel*). *d* and *e*, Dicer protein and mRNA expression were determined after overexpression of mutant p53 (*273H*) or a mutant p53 deletion construct (*273H* Δ*347*) in H1299 cells. The *left panels* show Dicer and p53 protein expression in a Western blot analysis with actin as a loading control. The *right panels* show relative Dicer mRNA expression. The *bottom panels* show the quantification of Dicer protein expression in three independent experiments corrected for actin expression with Dicer levels in control-transfected cells set to 1. *Error bars* indicate the S.E. of three experiments.

A number of mechanisms regulate Dicer expression, including the transcription factor TAp63. Several p63 binding sites were identified within the Dicer promoter, and TAp63 can drive the expression of Dicer to suppress metastasis in mice (6, 34). Although many cancer cells predominantly express the ΔNp63 isoform, only TAp63 is expressed in H1299 cells, making them a good model to examine the role of TAp63 in the regulation of Dicer expression in human cells. Depletion of TAp63 resulted in a reduction in Dicer expression (Fig. 3*a*). However, overexpression of TAp63α did not further increase Dicer expression (Fig. 3*b*), suggesting that although TAp63 may be required, it is not sufficient to promote enhanced Dicer levels in these cells. Depletion of TAp63 resulted in cell scattering similar to that seen following Dicer depletion (Fig. 3*c*). Because mutant p53 can function by inhibiting TAp63, we explored the possibility that mutant p53 might, via p63, reduce Dicer expression to drive invasion and scattering. Transient expression of mutant p53 273H in H1299 cells led to a dose-dependent decrease in the expression of endogenous Dicer protein and mRNA (Fig. 3*d*). Previously, we reported a role for the C terminus of mutant p53 in p63 inhibition (16), and a C-terminally truncated mutant p53 construct (d347) repressed Dicer expression to a lesser extent (Fig. 3*e*). These data suggest that mutant p53 can act at least partially through TAp63 to regulate Dicer expression. Despite the reproducibility of the effect of mutant p53 on Dicer levels, we noted that this response required the transient expression of mutant p53 to levels higher than those seen in the stable cell lines (Fig. 3*a*) or endogenous mutant p53 expression (Figs. 5 and 6). Dicer is induced in response to stress (35, 36), and these observations may therefore reflect a compensatory stress-induced increase in Dicer expression following the transient transfection of mutant p53.

**FIGURE 4.**
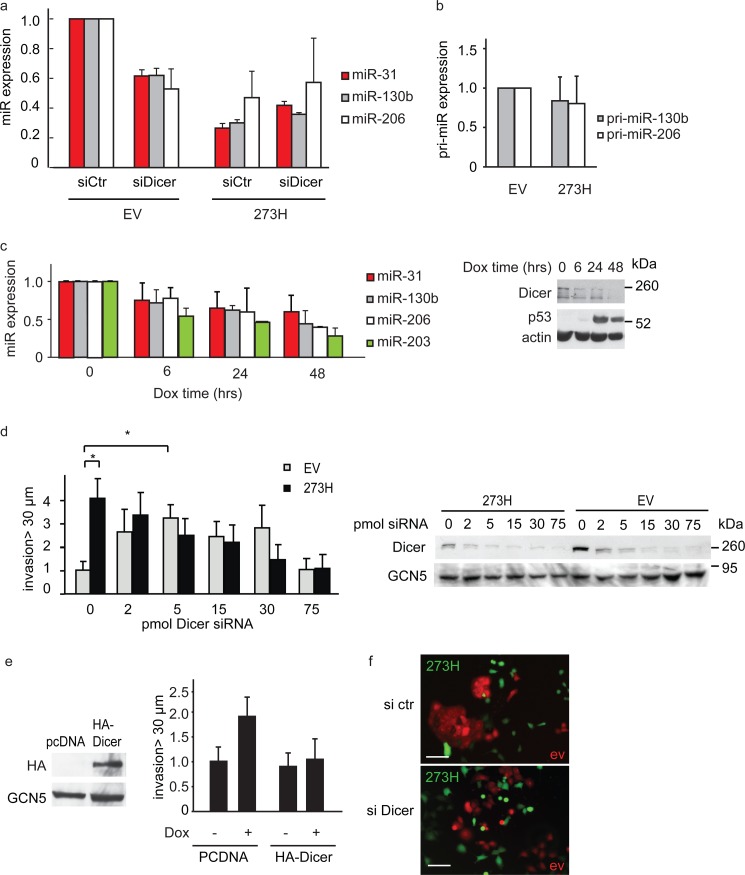
**Mutant p53 regulates the expression of mature miRs.** *a*, relative expression of mature miRs measured using qRT-PCR in control (*EV*) or mutant p53-expressing (*273H*) H1299 cells transfected with control or Dicer siRNA. Values are mean ± S.E. of three experiments. *b*, expression of the indicated pri-miRs was measured using qRT PCR in control or mutant p53-expressing H1299 cells. Values are mean ± S.E. of three experiments. *c*, relative expression of mature miRs was measured in time using qRT-PCR in control or doxycycline (*Dox*)-induced mutant p53 175H H1299 cells (*left panel*). Values are mean ± S.E. of three experiments. Dicer and p53 expression were verified by Western blot analysis with actin as a loading control (*right panel*). *d*, stable H1299 mutant p53 273H cells were transfected with increasing amounts of Dicer siRNA, and invasion was measured in Matrigel invasion assays (*left panel*). Values are mean ± S.E. of five experiments. Knockdown of Dicer was verified by Western blot analysis, and a representative Western blot of one of the experiments in the graph is shown. GCN5 was used as a loading control (*bottom row*). Also shown are the results from the control EV H1299 cells shown in Fig. 1*a* to allow for easy comparison with p53 273H H1299 results. * indicates statistical significant changes (*p* < 0.05 in a Student's *t* test). *e*, invasion of doxycycline-induced mutant p53 175H H1299 cells after overexpression of HA-Dicer or a vector control (*pcDNA*). Values are mean ± S.E. of three experiments. HA-Dicer expression was verified by Western blot analysis with actin as a loading control (*left panel*). *f*, control (*red*) or mutant p53 (*green*) cells were transfected with control or Dicer siRNA. Microscopic fluorescent images were taken to show scattering. *Scale bars* = 50 μm.

Dicer and/or TAp63 have been shown to regulate several miRs that play a role in invasion, including miR-31, miR-203, miR-130b, and miR-206 (6, 24). As expected, loss of Dicer decreased the expression of these mature miRs (Fig. 4*a*). A similar decrease was found in mutant p53 273H-expressing cells (Fig. 4*a*) and in H1299 cells that inducibly expressed another TAp63-inhibiting p53 mutant, 175H (*c*). Moreover, decreased Dicer expression in mutant p53-expressing cells did not further suppress the expression of miRs (Fig. 4*a*), and the expression of primary miRs was not affected by mutant p53 expression (*b*). Notably, many of these miRs are not exclusively regulated by TAp63 (*e.g.* miR-206 can be regulated by NRF2 and miR-31 by Runx2) (37, 38), possibly explaining the lack of further down-regulation of miR-206 after mutant p53 expression or the slight up-regulation of some miRs in mutant p53 cells after knockdown of Dicer. Together, these results support the hypothesis that mutant p53 prevents the maturation of pre-miRs by inhibiting Dicer expression.

We next investigated the effects of the loss of Dicer on invasion and scattering of mutant p53 cells. Loss of Dicer in mutant p53 cells did not further promote invasion (Figs. 4*d* and 1*a*), and overexpression of Dicer decreased the invasion of doxycycline-induced mutant p53 cells (Fig. 4*e*). These data indicate that mutant p53 acts through Dicer to promote invasion. Notably, invasion of mutant p53 cells showed a dose-dependent decrease in invasion upon Dicer knockdown, reflecting the threshold effect of Dicer expression required for invasion as described for Fig. 1*a*. Furthermore, coculturing Cherry-tagged control H1299 cells with GFP-tagged mutant p53-expressing H1299 cells showed clearly that control cells grow in colonies, whereas the expression of mutant p53 promoted a more dispersed growth pattern (Fig. 4*f*). Partial loss of Dicer resulted in a scattering of control cells but did not noticeably influence the dispersed pattern of mutant p53 cells (Fig. 4*f*).

##### Endogenous Mutant p53 Regulates TAp63 and Dicer in MDA MB231 Cells

**FIGURE 5.**
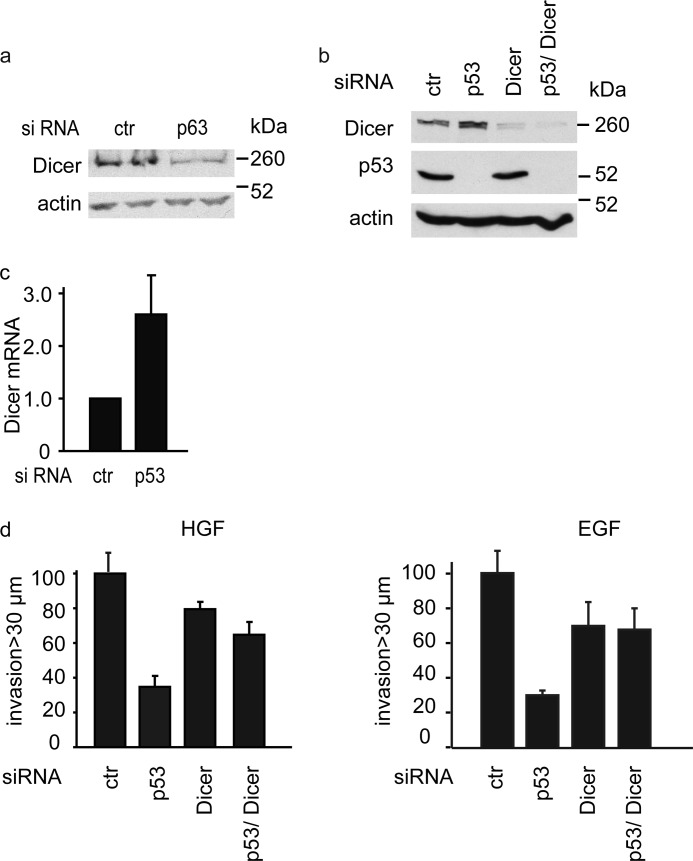
**Endogenous mutant p53 inhibits Dicer to promote invasion.** *a*, MDA MB231 cells were transfected with p63 siRNA, and Dicer expression was assessed by Western blot analysis using actin as a loading control (*ctr*). *b* and *c*, Dicer protein expression (*b*) or mRNA expression (*c*) of MDA MB231 cells transfected with p53 siRNA was monitored by Western blot analysis or qRT-PCR. Actin was used as a loading control. Values are mean ± S.E. of three experiments. *d*, the invasion of MDA MB231 cells toward HGF (*left panel*) and EGF (*right panel*) was determined. Values are mean ± S.E. of three experiments.

To confirm our results, we used MDA MB231 tumor cells, which express both TAp63 and endogenous mutant p53. Depletion of TAp63 reduced Dicer levels (Fig. 5*a*), whereas, conversely, siRNA knockdown of mutant p53 resulted in an increase in Dicer expression at the protein and mRNA levels (Fig. 5, *b* and *c*). MDA MB231 cells also invaded toward HGF and EGF in a mutant p53-dependent manner (Fig. 5*d*). Although depletion of Dicer resulted in a slight reduction of invasion (Fig. 5*d*), knockdown of Dicer and mutant p53 rescued the loss of invasion seen following mutant p53 depletion (*d*). These results support a model in which the ability of mutant p53 to promote invasion reflects a p63-dependent regulation of Dicer.

##### Endogenous Mutant p53 Regulates Dicer in a TAp63-independent Manner in HT29 Cells

**FIGURE 6.**
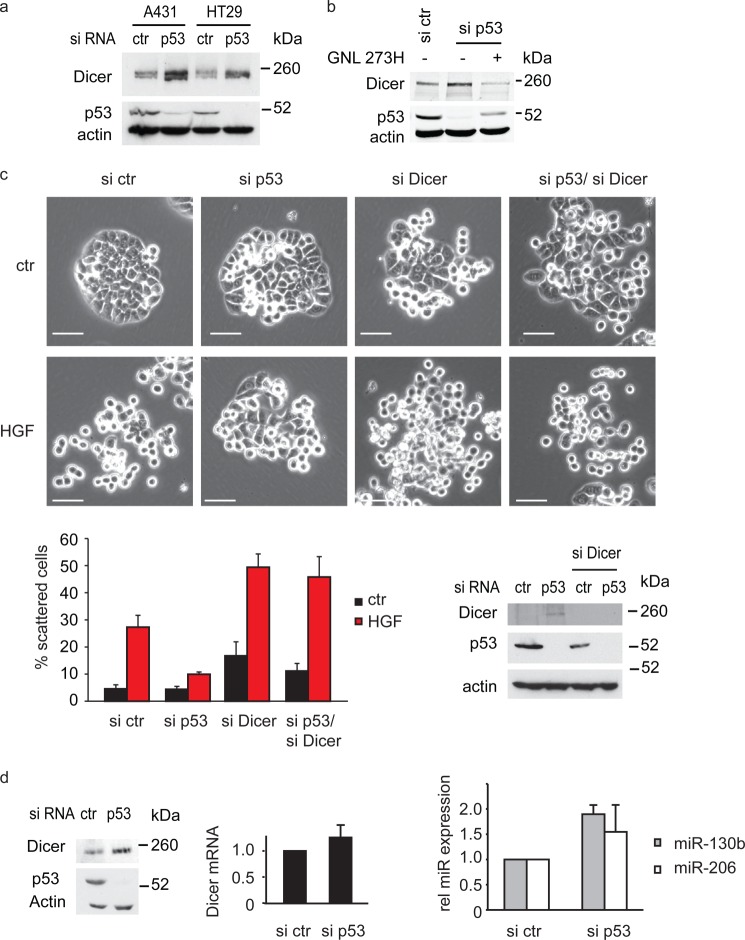
**Endogenous mutant p53 can inhibit Dicer in a p63-independent manner to promote scattering.** *a*, A431 or HT29 cells were transfected with p53 siRNA and monitored for Dicer and p53 expression by Western blot analysis. Actin was used as loading control (*ctr*). *b*, HT29 cells were transfected with an siRNA resistant mutant p53 (GNL 273H) construct in combination with p53 siRNA. Expression of Dicer and p53 was determined by Western blot analysis using actin as a loading control. *c*, scattering of HT29 cells transfected with p53 and/or Dicer siRNA in response to 10 ng/ml HGF (*top panels*). Scattering was quantified, and values are mean ± S.E. of three experiments (*bottom left panel*). Knockdown of p53 and Dicer was verified by Western blot analysis with actin as a loading control (*bottom right panel*). *Scale bars* = 50 μm. *d*, HT29 cells were transfected with control or p53 siRNA, and Dicer protein (*left panel*) or mRNA (*center panel*) expression was determined using Western blot analysis or qRT-PCR. p53 knockdown was verified by Western blot analysis, and actin was used as a loading control (*left panel*). Relative miR expression was determined using qRT-PCR (*right panel*). Values are mean ± S.E. of three experiments.

Although TAp63 appears to play a role in the regulation of Dicer expression, many cancer cells express either barely detectable levels of p63 (such as HT29 cells) or predominantly express ΔNp63 (such as A431 cells). Because both HT29 and A431 cells also express mutant p53 (273H), we explored the role of mutant p53 in regulating Dicer protein expression in these cells. Despite the lack of detectable TAp63 expression, knockdown of mutant p53 in either HT29 or A431 cells resulted in an increase in Dicer levels (Fig. 6*a*). In HT29 cells, this increase in Dicer levels could be prevented by ectopic expression of siRNA-resistant mutant p53 (GNL273H) showing siRNA specificity (Fig. 6*b*). HT29 cells grew in colonies under normal tissue culture conditions but scattered in response to HGF treatment (Fig. 6*c*). Depletion of the endogenous mutant p53 prevented this scattering, indicating that, in these cells, mutant p53 also sensitizes cells to HGF-induced scattering. Interestingly, depletion of Dicer by siRNA rescued the ability of HGF to drive scattering in mutant p53 knockdown cells (Fig. 6*c*). These data suggest TAp63-independent effects of mutant p53 on Dicer expression and are consistent with our previous studies showing that depletion of p63 did not change the sensitivity of either A431 or HT29 cells to scattering and did not reverse the inhibition of scattering seen upon depletion of mutant p53 (23). Interestingly, the changes in Dicer protein levels were accompanied by an increased expression of miR-130b and miR-206, although there was no change in Dicer mRNA expression (Fig. 6*d*), suggesting that the TAp63-independent effects of mutant p53 on Dicer expression are not mediated by transcriptional regulation.

## DISCUSSION

Our data demonstrate a role for Dicer in limiting invasion and scattering through the regulation of RCP-dependent recycling of integrins, EGFR, and MET. A partial loss of Dicer increased the recycling of these receptors, promoted RCP recruitment to α-5 integrin, and induced a more pronounced activation of EGFR and MET upon ligand stimulation. Taken together, these data point to a central role for miRs in programming the function of receptor recycling to drive invasive migration. There are many components of the endocytic transport and sorting machinery that are targets of miRs (39–42), and future work will be necessary to determine how the microRNA products of Dicer can specifically control trafficking of integrin and receptor tyrosine kinases (RTKs) to promote invasion.

In accordance with various recent publications, we could also demonstrate a role for mutant p53 in regulating miR expression (24–27). Interestingly, the majority of the published mutant p53-regulated miRs are down-regulated, supporting our observation that mutant p53 can inhibit Dicer function. In addition, Suzuki *et al.* (43) showed a role for wild-type p53 in promoting the maturation of miRs via interaction with the microRNA processing complex, DDR8/Drosha. In concordance with our results, the authors demonstrated a decrease in the maturation of miRs after mutant p53 expression, which coincided with a loss in the ability of mutant p53 to interact with DDR8/Drosha, although the precise mechanisms underlying the mutant p53-dependent deregulation of miRs were not investigated (43). These results suggest that both wild-type and mutant p53 have prominent but opposing roles at various levels in the process of miR maturation.

Many important biological processes are regulated through different but parallel mechanisms in mammalian cells. Our results show for the first time that mutant p53 can function through at least two different mechanisms to interfere with Dicer function: in a transcriptional manner through TAp63 and in a transcription- and TAp63-independent manner. Interestingly, recent data by Shen *et al.* (44) are suggestive of a possible third regulatory mechanism. The authors show that EGFR can interact with a microRNA machinery component, AGO2, in response to hypoxia (44), a condition that causes a retention in EGFR in endocytic trafficking compartments (45). Binding of EGFR to AGO2 reduced the binding of AGO2 to Dicer, resulting in reduced miR maturation (44). As mutant p53 enhances EGFR trafficking and signaling (16), it will be interesting to see whether mutant p53-induced EGFR activation can also interfere with Dicer function and miR maturation.

Besides regulating invasion and metastasis, mutant p53 promotes various other processes. Some of these are dependent on the inhibition of TAp63, but others reflect the ability of mutant p53 to regulate other proteins and transcription factors such as SREBPs, NF-Y, SP-1, or VDR (20, 46–48), through which mutant p53 can promote cell proliferation, chemoresistance, cholesterol metabolism, and various other tumor-promoting processes. A large number of genes that regulate these processes are targets of miRs. Loss of Dicer has been associated with decreased chemosensitivity and cell proliferation in ovarian cancers (49) but, conversely, also with increased cisplatin sensitivity and cell proliferation in MCF-7 breast cancer cells (50). This discrepancy could suggest tumor-specific Dicer functions but could also be a reflection of a threshold effect of Dicer concentration, as was observed by us (Figs. 1*a* and 4*d*) and others (11), in which an optimal concentration of Dicer was required for the promotion of invasion. As p53 is among the most frequently mutated proteins in cancer, it will be important to explore the role of mutant p53-dependent (and p63-independent) Dicer regulation in all gain-of-function activities of mutant p53.

## Footnotes

2 The abbreviations used are:

miR: microRNA
pri-miR: primary miR
pre-miR: precursor miR
TA: transactivation
RCP: Rab coupling protein
EGFR: EGF receptor
HGF: hepatocyte growth factor
qRT-PCR: quantitative PCR
EV: empty vector.
